# Predictions of household water affordability under conditions of climate change, demographic growth, and fresh groundwater depletion in a southwest US city indicate increasing burdens on the poor

**DOI:** 10.1371/journal.pone.0277268

**Published:** 2022-11-23

**Authors:** Josiah M. Heyman, Alex Mayer, Jessica Alger

**Affiliations:** 1 Department of Sociology and Anthropology, University of Texas at El Paso, El Paso, Texas, United States of America; 2 Department of Civil Engineering, University of Texas at El Paso, El Paso, Texas, United States of America; 3 Department of Civil & Environmental Engineering, Michigan Technological University, Houghton, Michigan, United States of America; The University of North Carolina at Greensboro, UNITED STATES

## Abstract

Reduced river flows and groundwater depletion as a result of climate change and population growth have increased the effort and difficulty accessing and processing water. In turn, residential water costs from municipal utilities are predicted to rise to unaffordable rates for poor residential water customers. Building on a regional conjunctive use model with future climate scenarios and 50-year future water supply plans, our study communicates the effects of climate change on poor people in El Paso, Texas, as water becomes more difficult and expensive to obtain in future years. Four scenarios for future water supply and future water costs were delineated based on expected impacts of climate change and groundwater depletion. Residential water use was calculated by census tract in El Paso, using basic needs indoor water use and evaporative cooling use as determinants of household water consumption. Based on household size and income data from the US Census, fraction of household income spent on water was determined. Results reveal that in the future, basic water supply will be a significant burden for 40% of all households in El Paso. Impacts are geographically concentrated in poor census tracts. Our study revealed that negative impacts from water resource depletion and increasing populations in El Paso will lead to costly and difficult water for El Paso water users. We provide an example of how to connect future resource scenarios, including those affected by climate change, to challenges of affordability for vulnerable consumers.

## Introduction

We propose that usable water is becoming more costly and difficult. The term “difficult” in this phrasing refers to the increased effort needed to access and process water. An example is desalination. “Costly” is a characteristic consequence of resorting to more difficult ways to supply water. These changes occur in the context of climate change, which in some cases reduces river flows. They also occur in the context of fresh groundwater depletion, meaning that nearby, inexpensive resources cannot meet demand. Water will not simply run out in most cases, but rather replacement supplies will be more costly and difficult.

There are some hints of this in recent trends in which water utility bills are rising faster than incomes. Costs associated with water and related municipal services have increased 2.5 times faster than overall increases in consumer expenses: the U.S.-average consumer price index (CPI) for Water and Sewer and Trash Collection Services has increased by 148% from 1998–2020, while the overall U.S.-average CPI has increased by 60% over the same period. Water rate surveys from the American Water Works Association of more than 60 water utilities reports indicate an average increase of 4% per year for water rates between 2008 and 2016 [1]. Circle of Blue [2] reports an average 5.2% per year increase in water bills for 30 US cities over a recent nine-year period (2010–2018). The US-city average consumer price index (CPI) for all items rose 69% between December 1997 and September 2021 [3], while the CPI for water, sewer, and trash services rose at more than twice times that rate (164%) over the same period [4]. The drivers of these price increases vary from utility to utility, but reports mention adopting new, more expensive water sources in response to climate variability or increased demand; increasing infrastructure replacement costs associated with deferred maintenance; more expensive treatment in response to emerging contaminants and regulatory requirements; and the shift from federal to local funding for water infrastructure projects [5–8]. We inquire into the impact of the water source component of these trends on the cost of residential water provided by municipal utilities.

The “costly and difficult” conceptualization contributes to a small but insightful literature on the cost of municipal water to poor household consumers in the United States. Mack and Wrase [6] and Colton [9] posit increasing cost stress on the poor in the future, because of climate change and aging infrastructure. However, their models of future water cost are linear projections from the recent past. While justified in terms of some recent cost drivers (e.g., infrastructure aging), they do not have a concrete model of future costs and specifically do not connect costs to longer term and more systematic biophysical trends, such as the need to acquire more expensive supplies because of climate change-linked reductions in water availability. We think those connections are important. To determine future costs to water consumers requires models of climate change and resource depletion, as well as major technology and resource transitions in response. These transitions do not occur simply as smooth projections of the recent past but tend to unfold when thresholds of water availability are reached. We use a regional conjunctive water use model coupled with future climate scenarios and detailed 50-year future water supply plans with expected water supply costs and volumes. Our work, building on Mack and Wrase [6], offers a new angle on how climate change and resource depletion might impact the poor through dramatic increases in the cost of municipal water supplies. We add to the widening literature on household water insecurity that identifies water affordability as a critical issue [10].

### Previous literature

Generally, researchers find that currently municipal water is affordable, with some exceptions, but they draw attention to challenges for low-income consumers [6,9,11–13]. Mack and Wrase [6 p1], for example, estimated that 11.9% of U.S. households would have combined water and sewer bills over 4.5% of household income. Teodoro [12 p18] does not take a national approach, but for the 25 largest cities he finds that for water and wastewater households in the bottom 20% of income average paid 11.4% of their income available after other necessary expenses. Among them, El Paso is relatively affordable, costing 6.9% of available income to the bottom quintile. Teodoro and Saywitz [13] uses a national data set, finding that the bottom 20% pay on average 9.7% of their available income for basic water and wastewater after other expenses. Colton [9] confirms the challenge of affording water in low-income census tracts in twelve cities. Water thus generally has been affordable up to the present day but is markedly more expensive relative to income for the very poor.

The main differences between these studies are methodological. Mack and Wrase [6], for example, examine the cost of the average volume consumed by an average household (that is, one with a median household income), even as they admit that water consumption has discretionary elements that might be affected by income differences, rising prices, and climate. Teodoro [12], on the other hand, examines a fixed, basic need amount of water (a minimum needed for indoor uses such as drinking, washing, and so forth), arguably more fundamental and less subject to variable patterns of usage. Teodoro also focuses attention on impacts of water cost on poor (more cost-vulnerable) rather than median-income households by focusing on the bottom quintile (20%) of incomes relative to costs. Colton [9] renders a similar analysis by looking at the Federal Poverty level income. In income-cost comparisons, Mack and Wrase [6 p6-7] suggest a standard that the water supply bill should be no higher than 2% of income and combined water and wastewater no higher than 4.5%. Teodoro [12] offers a relative impact alternative, percentage of income at the twentieth percentile income devoted to water. Finally, Mack and Wrase [6] and Colton [9] offer a more geographically fine-grained analysis by examining census tracts, which enables the analyst to identify geographically concentrated impacts of water bills, and likewise to provide richer analysis of social correlates of impact by using American Community Survey data available for census tracts, the smallest unit for which data is available. Teodoro only considers the entire city within which a utility is based. No one method is perfect but each of these offer helpful leads in designing models for discerning present and future cost impacts.

The existing literature on household water cost is limited in its approach to future trajectories. Mack and Wrase [6 p8, utilizing 8] extrapolate two recent past rates of change for household consumers, 2015 over 2014 (6%), and 2010–2015 (41%), as estimates of possible future change. Likewise, Colton [9 p33, utilizing 1] uses observed change from 2008 to 2016 for industrial scale water supplies (8-inch water meters) to project residential cost change to 2030 (4.71% annually). (Teodoro [12] does not examine the future.) While linear extrapolation from the past provides insights, it fails to consider future drivers of change, which arguably will accelerate change rather than following linear paths. These include climate change, depletion of easier and/or cheaper water sources, and future demographic change.

The wider water literature has moved to using scenarios to address non-linear, complex change. A “scenario” approach considers alternative choices among input models affecting supplies (e.g., climate change), demand change (e.g., demographics), and policy choices (e.g., groundwater depletion policies) [examples include 14–18]. These studies are increasingly seen as key for water utility planning [19–23]. Scenarios are not meant to be deterministic predictions of the future but rather offer a set of plausible futures and alternative choices.

## Materials and methods

### 3.1 Regional setting and modeling

We examine a major urban utility, El Paso Water (EPW), a public utility serving the city of El Paso, Texas and a few bordering areas outside the city. Our choice takes advantage of the utility’s 50-year planning data with water sources, volumes, and costs in present-value dollars, and likewise our recently developed model of the Paso del Norte basin, also going out 50 years. That model includes considerations of climate change, aquifer depletion, and complex regional governance and usage [24]. Through this robust case study, we are able to get past the limits of linear extrapolation in examining future water supply prices.

**Fig 1:**
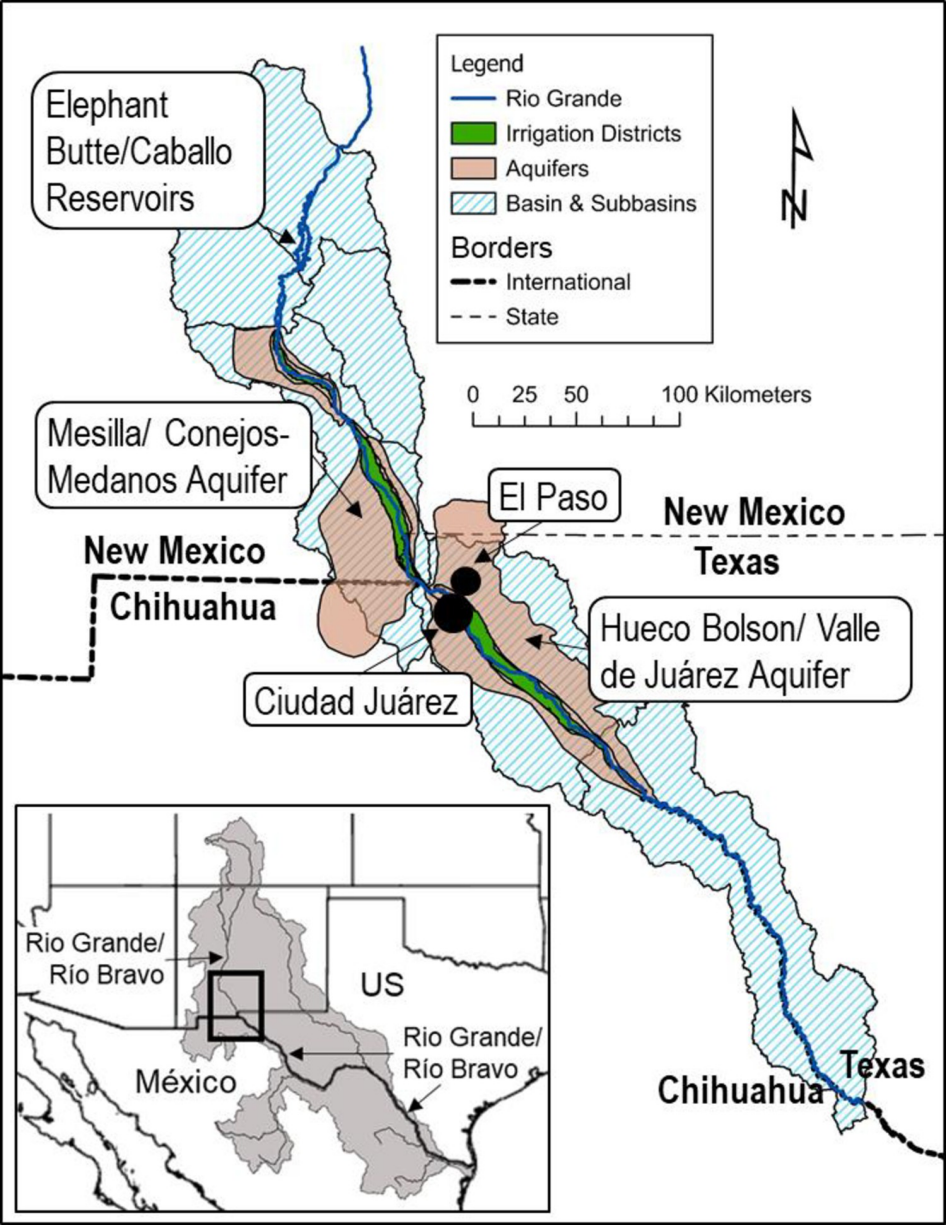
Study area map indicating major sources of water supply for El Paso Water and other users. Source: Hydrological features, work of the authors; basemaps, US Census.

The Paso del Norte basin consists of the Rio Grande (Río Bravo del Norte in Mexico) from Elephant Butte reservoir to the entrance of the Río Conchos (Fig 1) and two major aquifers, the Mesilla Bolson (Conejos Medanos in Mexico) and the Hueco Bolson (Valle de Juárez in Mexico). The Rio Grande is mainly fed, in this reach, by snowpack melt in southern Colorado and far northern New Mexico. The headwaters snowpack is shaped by climate. The river delivery into the Elephant Butte reservoir is set by interstate compact as a portion of that climate-driven headwaters output. The annual release from Elephant Butte is the main regional source of surface water, although variation in local precipitation (mean annual precipitation = 8.67 in, 1938–2019) has modest effects. Hence the river input into the controlling reservoir is the main path by which climate scenarios shape surface water supplies. Because El Paso Water leases significant rights to surface water from farmers, its water supply is affected by climate change scenarios that our regional models indicate will tend to reduce river supplies (“river drought”).

The conjunctive alternative to surface water in this region is water pumped from the Mesilla Bolson and the Hueco Bolson. The aquifers are pumped by the large Mexican city, Ciudad Juárez (population = 1,512,450 in 2020 [25]), the large U.S. city, El Paso (population = 681,728 in 2019 [26]), smaller municipal utilities in El Paso county, and agriculturalists in both countries. EPW uses both basins, though it relies more on the Hueco Bolson. The aquifers are being pumped well beyond their modest recharge rates; very old residence times for large, urban wells indicate on-going depletion [27].

Both aquifers contain fresh and brackish water, with the division between fresh and brackish water demarcated when total dissolved solids (TDS) roughly equal to 1,000 mg/L. In a non-drought, full river-allocation year, EPW water sources consist of 40% Rio Grande water, 38% Hueco Bolson freshwater, 17% Mesilla Bolson freshwater, and 5% desalinated Hueco Bolson brackish water. In a characteristic drought year, sources consist of 7% Rio Grande water, 61% Hueco Bolson freshwater, 27% Mesilla Bolson freshwater and 5% desalinated Hueco Bolson brackish water. As these numbers indicate, EPW attempts to limit aquifer depletion when it can but river drought within current supply sources and technologies pushes the utility to extract more from the aquifer [27]. Hence, another component of our scenario modeling of the future is the timing of depletion of freshwater in local aquifers.

We are part of a wider project that has created a water balance model designed to provide various future scenarios based on different drivers (available at https://swim.cybershare.utep.edu/en/home, [24]). The model simulates snowpack melt-driven river input to Elephant Butte and Caballo reservoirs, based on alternative climate scenarios; reservoir evaporation and releases; watershed runoff from local precipitation, river channel-aquifer interactions; conjunctive groundwater extraction in each aquifer by agriculturalists (based on fluctuating surface water availability); urban groundwater extraction by other utilities besides EPW; conjunctive groundwater extraction by EPW (again based on fluctuating surface water availability); and recharge to the aquifers. To already published results from this project, in which urban water economic values are treated in a limited fashion vis-à-vis alternative sectors, we add a more specific model of changing supplies in El Paso, and then a model of water supply cost changes. Overall, we have a dynamic system model, capable of producing many alternative multi-year scenarios, which gives us a powerful tool for examining future water costs.

### 3.2 Future water demand and supply scenarios

**Fig 2:**
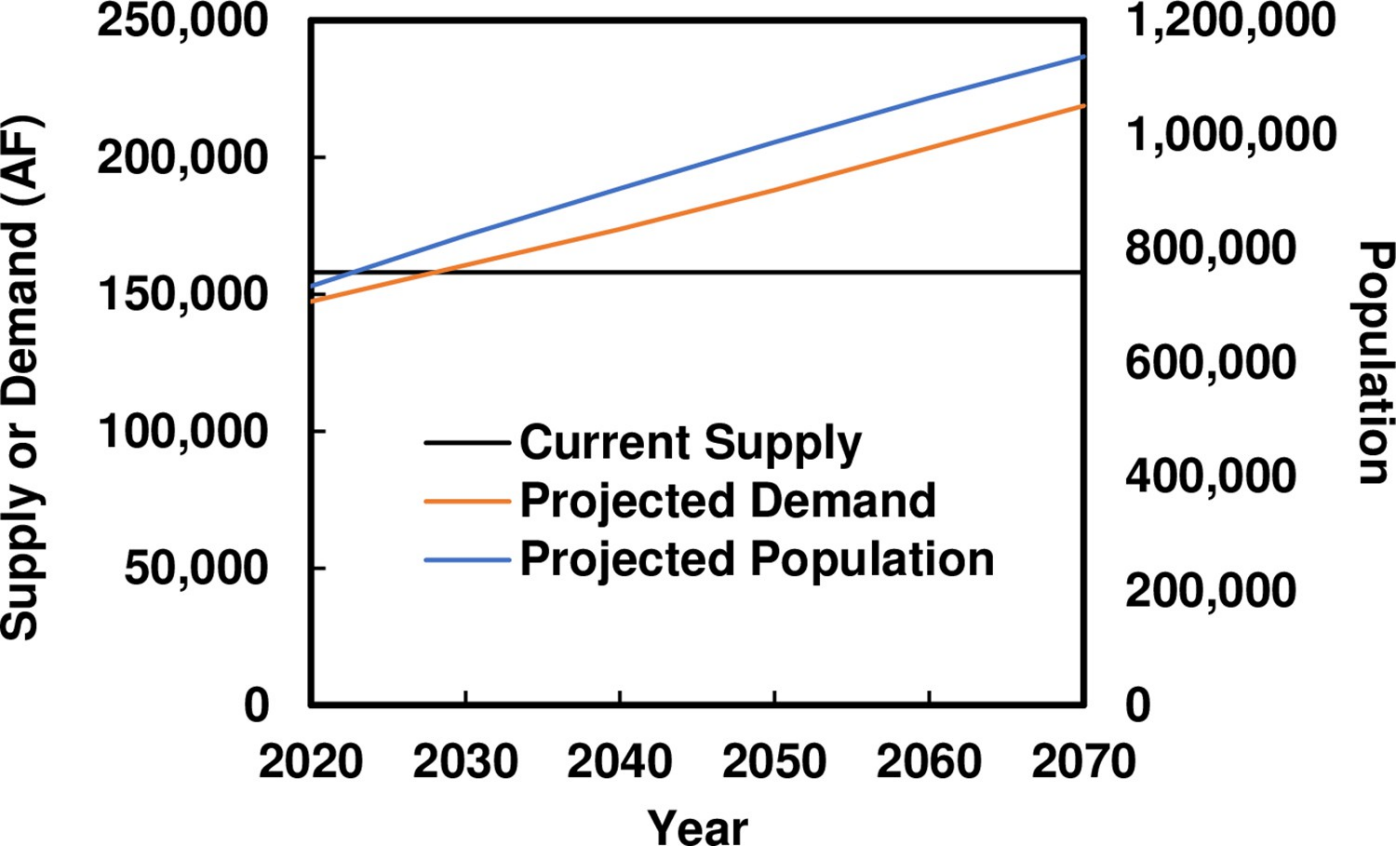
Projected annual water supply and demand and population for the El Paso Water service area from 2020–2070. The current average annual water supply volume is shown for reference. Source: Work of the authors.

Projected water demands and water supply sources/costs for 2020 to 2070 for EPW were taken from the 2016 Far West Texas Water Plan (FWTWP) [28]. Water Plans for the state of Texas provide regional evaluations of future water demands, currently available supplies, and new supply and demand strategies for meeting future water shortages. Fig 2 shows FWTWP projected populations and water demands and estimates of water availability from current water supply sources. Water demand is expected to increase by 48% from 2020 to 2070. As a result, deficits between current supply and projected demand are expected to be as high as 88,000 acre feet (AF) in 2070. Over the same period, population is expected to increase by 55% in the EPW service area. The rate of increase in demand is lower than the rate of increase in in population because a relatively small (6%) decrease in per capita use is predicted in the FWTWP projections, due to conservation measures.

The FWTWP projects future water supply availability from existing sources, plans for new sources, and reductions in per capita demand, to compensate for the deficit between projected demand and current water supply availability indicated in Fig 2. Table 1 lists the expected volumes for existing and new sources identified in the FWTWP, which we refer to as the “Base Case” water supply scenario. Of note in Table 1 is that (a) importation of groundwater from new, more distant aquifer sources is expected to begin in 2030 and gradually increase, (b) use of Hueco Bolson aquifer freshwater is expected to decline by roughly 6% every 10 years, to be compensated for by increases in desalinated Hueco Bolson aquifer brackish water and (c) Rio Grande supplies are expected to be constant.

**Table 1:** Annual water supplies by source from 2016 Far West Texas Water Plan [28]. kAF = thousands of acre feet per year.

	Supply (kAF/yr)					
Source	2020	2030	2040	2050	2060	2070
Mesilla aquifer freshwater	27	27	27	27	27	27
Hueco Bolson aquifer freshwater	60	57	54	50	47	44
Rio Grande	63	64	65	66	66	67
Desalinated Hueco Bolson aquifer brackish water	8	11	13	16	18	21
Imported groundwater	0	5	11	16	22	27
Advanced wastewater purification and aquifer recharge	0	5	9	14	18	23
Total	158	168	179	189	200	210

However, we suggest that the FWTWP estimates of water availability from two primary current sources, Hueco Bolson freshwater and the Rio Grande, are overly optimistic. First, given current freshwater pumping rates from the Hueco Bolson aquifer by El Paso, Ciudad Juárez, and agricultural irrigators (approximately 188,000 AF/yr together), estimates of aquifer recharge rates (approximately 33,000 AF/yr), and remaining freshwater storage in the aquifer (approximately 6,500,000 AF), freshwater is expected to be completely depleted in 42 years [27]. It is likely that pumping rates in Ciudad Juárez will increase substantially in the meantime, as populations in this city may increase as much as 1.02% per year, for a total increase of 66% by 2070. Given current aquifer recharge rates and expected increases in pumping by Ciudad Juárez, we estimate, using our water balance model (https://swim.cybershare.utep.edu/en/wb-intro), that EPW’s current rate of pumping of freshwater from the Hueco Bolson will need to be reduced from the amounts predicted in the FWTWP by 50% by 2070 to extend the aquifer life past 2070.

Second, climate change in the headwaters of the Rio Grande is expected substantially to decrease downstream supplies, with releases from Elephant Butte reservoir projected to decline 10% in 2021–2070 than in 1971–2020 [29]. Using a pessimistic climate change scenario associated with the Access85 climate model projection, inflows into Elephant Butte will decline by 39% on average over the period 2020–2070, effectively reducing average water availability for EPW from the Rio Grande by the same fraction. Using projected annual Elephant Butte reservoir inflows from Townsend and Gutzler [30] for the Access85 climate model projection, the declines in Rio Grande water availability by year will be 37%, 56%, 60%, 41%, and 23% in the years 2030, 2040, 2050, 2060, and 2070, respectively. These declines were calculated using the water balance model.

Four scenarios for future supply volumes by source were determined based on the expected impacts of climate change and groundwater depletion and how these decreases will be compensated by increases in desalinated Hueco Bolson brackish groundwater versus imported groundwater. While depletion of Hueco Bolson fresh groundwater is expected to occur in a matter of a few decades [27], less is known about the availability of Hueco Bolson brackish groundwater over the next 50 years. To contend with this uncertainty, we incorporate into the scenarios the possibility that reductions in Rio Grande supply and Hueco Bolson freshwater pumping will be compensated by either 100% from desalinated Hueco Bolson brackish groundwater or 100% from imported groundwater.

All four scenarios involve meeting the demand projected in Fig 2. The four water supply scenarios are: (1) “Base Case,” which is based the FWTWP expectations for meeting supply deficits; (2) “Climate Change + Desalination,” in which the Rio Grande water supply is reduced according to the Access85 climate model predictions and the reduction is compensated by increasing desalinated brackish groundwater from the Hueco Bolson; (3) “Climate Change + Imported GW,” in which the Rio Grande water supply is reduced according to the Access85 climate model predictions and the reduction is compensated by increasing imported groundwater; and (4) “Climate Change + Imported GW + Reduction in HB Pumping,” in which the Rio Grande water supply is reduced according to the Access85 climate model predictions, freshwater pumping from the Hueco Bolson (HB) is reduced by 50%, and the two supply reductions are compensated by increasing imported groundwater. While many other scenarios can be generated, these capture the range of stresses and responses from least to greatest volume of transition in supplies. S1 Table in S1 File shows the annual volumes for each water source by scenario and by year.

### 3.3 Future water cost scenarios

**Fig 3:**
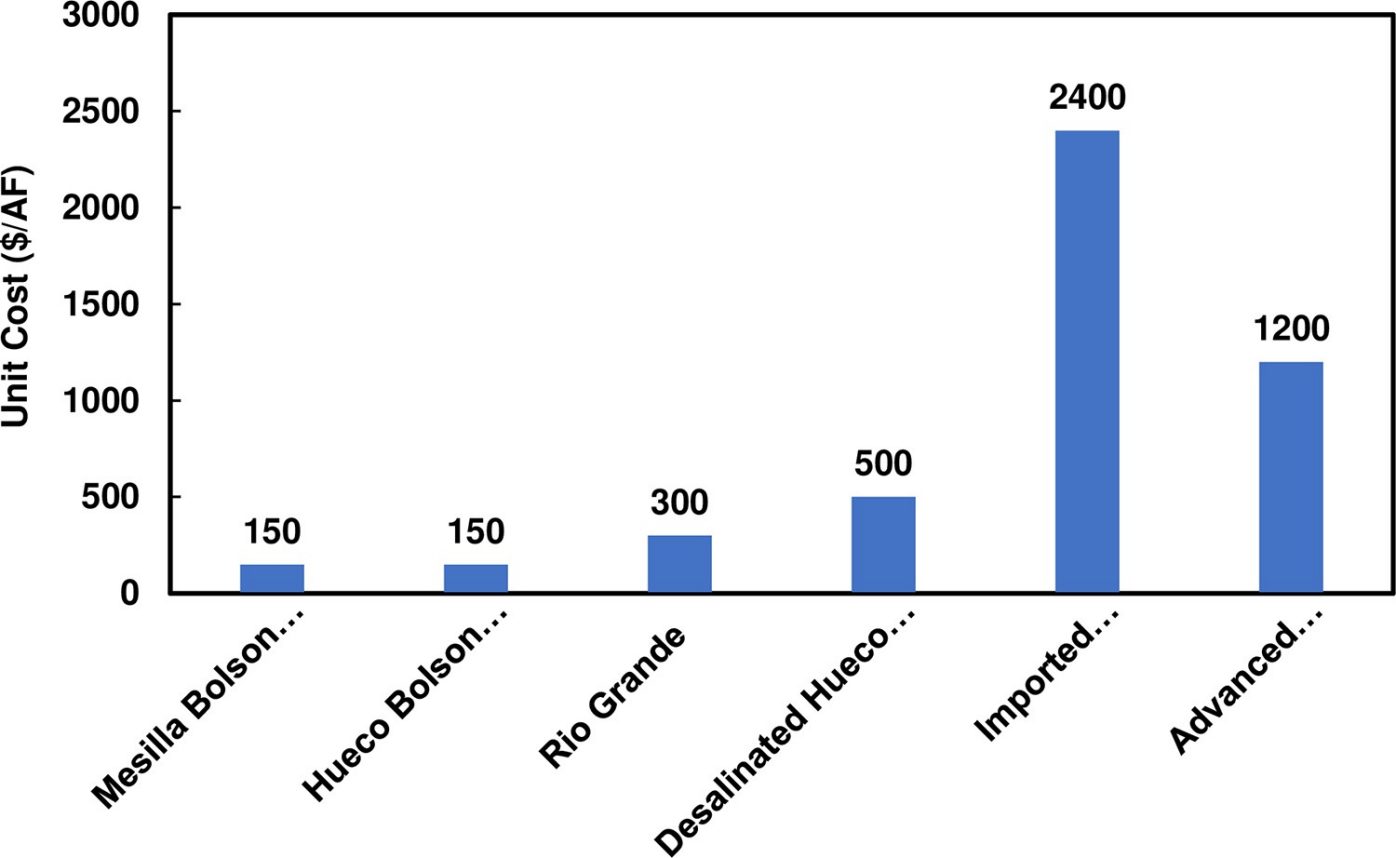
Annualized unit costs for water supply sources used in future supply and demand scenarios. Data source: Lisa Franklin Rosendorf, personal communication, El Paso Water, February 12, 2020. Graphic representation source: Work of the authors.

The 2016 FWTWP identifies costs of expansion of water supply sources. However, we updated costs of sources directly from EPW (Lisa Franklin Rosendorf, personal communication), which are summarized in Fig 3. These supply unit costs are per volume of water supplied and include amortized capital and operation and maintenance (O&M) costs. EPW gives dollar amounts for these costs in real 2020 dollars. To determine water supply costs every 10 years from 2020 to 2070 for each scenario, the unit costs in Fig 3 are multiplied by supply volumes for the respective sources in each of the four scenarios to obtain annual costs associated with each source. The annual costs for each water source are summed over the sources to obtain the total future water supply costs by year (see S2 Table in S1 File).

Our model focuses exclusively on changing supply mixes according to future supply and demand scenarios. Changes in the supply mix will affect prices, if costs stated in EPW planning documents are transmitted to consumers (a key assumption). However, we do not project any other changes in water prices; we follow the simplest assumption that all other costs remain equal to the rate of inflation (consumer price index)—maintaining the same real prices. Scientifically, simple assumptions reduce the bias from assumption choices. Our framework focuses the inquiry exclusively on future supply source, technology, and volume changes, a crucial topic in the current socio-environmental juncture. It may well be that other utility costs will change in ways different from the underlying rate of inflation or the incomes of the poor, but we do not have fifty-year models of those changes, only linear projections from the recent past. This is probably a conservative assumption, given recent rate increases (discussed above), but conservative assumptions are desirable in rendering a compelling case, when we note strong effects even with cautious assumptions.

### 3.4 Current and future residential water rates

Table 2 shows the EPW rate structure for residential water consumption in 2020. We exclude other charges on the water bill, such as wastewater, stormwater, and solid waste charges. The fixed costs in Table 2 are for meter sizes of 1 inch because we are focused on typical house sizes in El Paso, which tend to have meter sizes 1 inch or less. The tiered volumetric charge structure is based on an increasing block rate framework. As shown in Table 2, the determination of which tiers a given household falls under is based on multiples of the monthly average winter consumption (AWC), which is the average amount of water used during the most recent, winter billing periods in December, January and February. Because we don’t know the AWCs for individual residences, we use the default approach used by EPW, where the AWC is established by estimates of the average AWC by meter size. In the case of meter sizes of 1 inch, the AWC = 2992 gallons/month.

**Table 2:** El Paso Water residential monthly fixed and volumetric water charges for water in 2020 (Source https://www.epwater.org/customer_service/rates_and_fees).

Charge	Cost ($)	Basis
Water Supply Replacement Charge ^a^^,^^b^	11.59	per household
Monthly Minimum Charge ^a^	7.82	per household
Block 1—Over 2,992 gallons to 150% of AWC ^c^	0.31	per 100 gallons
Block 2–150% to 250% of AWC^c^	0.75	per 100 gallons
Block 3—over 250% of AWC ^c^	1.07	per 100 gallons
Franchise Fee ^a^	1.24	per household

^a^ meter size less than 1 inch.

^b^ applied if monthly household use ≥ 2,992 gallons.

^c^ AWC = average winter consumption.

Changes in future water rates are calculated by multiplying the flat and volumetric water charges from the 2020 EPW rate structure in Table 2 by the ratio of future total water supply costs (section 2.3) to current (2020) costs. These annual cost ratios are then adjusted to account for increases in numbers of households in the EPW service area, where it assumed that (a) the number of households will increase at the same rate as the population (Fig 2) and (b) the costs will be spread equally among households in the EPW retail service area. This simple approach assumes that EPW passes along supply costs directly to residential customers within the existing block tiered rate structure given in Table 2. The increasing block rate structure (lower per unit charges for low volumes, higher per unit charges for volumes above that base) favors lower volume, presumably poorer residential consumers. Hence, our conclusions about water cost impacts on poor consumers are conservative, in that we build in the current, relatively favorable pricing policy. If lower-volume consumers’ costs are frozen or further reduced, some income source needs to make up the difference in revenue to the water utility (a policy we support; see [31]). Our model then is also a step in this direction, as it allows scenario estimates for the cost of future adjustments to protect the poor.

### 3.5 Household data by census tract

Household and population demographic data for the five-year sample ending in 2019 was obtained for 137 census tracts within the EPW retail service area (see S3 Table in S1 File for census tract codes) from the US Census Bureau’s American Community Survey. (Newly released 2020 census tract boundaries differ from the 2010-vintage tracts used in our map of the EPW service area; to maintain consistency, we use the five-year ACS sample ending in 2019.) It included number of households, mean household size, household mean and median income, and the mean of each household income quintile. EPW supplied a service area map, which we laid over a map of census tract boundaries. We visually examined land cover maps for each of the 23 tracts bisected by a census tract boundary. We determined that the majority of the built-up areas in all 23 of these tracts were within the EPW service area boundaries and thus included all of the households in these tracts in our analysis.

We maintain 2019 incomes as constant real dollar incomes into the future (nominal income in each census tract and the utility district as a whole matches the rise in the consumer price index). A fifty-year model of social-economic indicators down to the household scale does not exist comparable to the one we build for water supply, and we are cautious about linearly projecting recent past trends across this long-time span. Again, maintaining 2019 real incomes with no speculative adjustments is a cautious assumption that has the virtue of not favoring our case. It helps to isolate the cost effects of changing supply mixes, the only modeled variable that differs from the consumer price index.

We follow Teodoro [12] in focusing on the impact on the poor by looking at income quintiles, in our case the bottom two quintiles. The main concern is the bottom quintile (20%) incomes but for some elevated cost scenarios we examine the bottom two quintiles (40%). Like Teodoro, we provide data for cost to the bottom quintile of the entire utility coverage area. We add a methodological insight from Mark and Wrase [6] and Colton [9] by adding a finer grained geographical approach to detect concentrated impacts, using income figures (quintiles) from individual census tracts inside the service area. The mean income of the lower quintiles in these tracts, and thus their ability to pay, is significantly lower than quintiles across the EPW service area as a whole. Tract by tract estimates, then, reveal important distributive environmental justice considerations.

No specific human participants were involved in this study.

### 3.6 Household water consumption

Following Teodoro [12], we model basic needs household water consumption as requiring 50 gallons per capita per day (gpcd), plus we add an allowance for evaporative cooler use for air conditioning. The 50 gpcd figure constitutes a minimum indoor water use, according to TWDB [32 p33]. The minimum indoor water use is scaled to the household level by multiplying by the average number of persons per household in a given census tract. The minimum indoor water use is assumed to remain constant throughout the year. For air-conditioning, we use an estimate of average water use per household for evaporative coolers in El Paso from Alger et al. [33] of 2,219 gallons per month. The evaporative cooler use is added to the minimum household indoor water use for the hot weather months of May through September. Evaporative coolers are less expensive than refrigerated air conditioning and represent a realistic addition to basic water needs for the poor of this region. These figures are geographically specific to Texas, and within it, to the dry desert region with evaporative cooling.

Our approach focuses on environmental justice. When we offer model results of future water supply costs to poor consumers of over five, sometimes even ten percent of income, it is important to keep in mind that this is the cost just for a basic human need for drinking, washing, and withstanding heat. While El Paso Water does not make available data on residential use by household income or census tract, this basic need amount of water (50 gpcd for eight months a year, and approximately 75 gpcd for four months a year with evaporative cooling) is significantly lower than the figure EPW does provide, total utility-supplied gallons per day divided by total service area population, which was 128 gpcd in 2018 [34].

Using a fixed, irreducible amount simplifies the modeling task, again reducing assumptions and biases. We do not include change in per capita consumption in response to increases in water prices, due to lack of price elasticity models going out fifty years; anyway, a fixed basic needs volume is likely to be inelastic. More prosperous consumers might conserve discretionary uses (e.g., outdoor plant watering, washing cars, etc.), but the basic need assumption removes discretionary uses from consideration specific to the cost of water supply impacts on the poor. Total urban consumption (i.e., if prices drive declining outdoor demand for the non-poor) affects the total EPW water supply needs in the water balance model, affecting our model of changing supply sources and costs; to address this effect, we follow the Far West Texas planning document [28]. The basic needs are mainly indoors uses; evaporative cooling affects the indoor environment though the cooler unit is located on the outdoor side of a dwelling. Outdoor demand, subject to discretion and climate change, is removed from the model. As discussed below, numerous studies show indoor water use is much more inelastic than outdoor use. In other words, we are not addressing the impact of future supply-driven cost changes on all consumers or average consumers, and not on indoor or outdoor, but rather we are extending the line of inquiry from Mack and Wrase, Teodoro, Colton, etc. about cost of a bare minimum of water for the poor, bringing a stronger examination of environmental change (supply transitions, specifically).

### 3.7 Calculation of fraction of household income spent on water consumption

The basic household water consumption for each census tract by month (Section 3.6) is compared to the EPW water charge rate structure (Table 2), to determine whether the water supply replacement charge should be applied and which block should be used for the volumetric charge. These two charges are added to the fixed charges for monthly minimum charge and the franchise fee. The total monthly charges are summed over the year, to obtain the base year (2020) total annual household expenditures for water consumption by census tract. As described in Section 2.4, the water charges in Table 2 are multiplied by the ratio of future water supply costs to the present (2020) water costs for the four water supply scenarios, by year, and the procedure for obtaining the annual household expenditures for water consumption by census tract is repeated for each scenario and year. The annual household expenditures for water consumption are then divided by mean household income for each quintile in each census tract (the mean for each quintile is what is available in the ACS; the median of the quintile is not reported). Our results, then, report the calculation of the fraction of household income spent on water consumption (FIWC), by household income quintile, in each census tract, and for each year in the four future water supply scenarios. The number of households in each income range (and associated representative income) and in each census tract is used to determine the frequency of households with given ranges of FIWC by income range and census tract.

In doing income-cost comparisons, Mack and Wrase [6 p6-7] suggest a standard that the water supply bill should be no higher than 2% of income (and combined water and wastewater no higher than 4.5%). Teodoro [12] offers a relative impact alternative, percentage of discretionary income at the twentieth percentile income devoted to water. We combine the best of these approaches. We report the percentage of income expended for the two lower income quintiles, and also indicate proportion of households surpassing a series of thresholds (2.5%, 5%, 7.5%, 10% of income).

The previous literature includes wastewater charges in bills as well as water service and supply. We agree—people pay the total bill, not just the water supply bill—but our environmental models of the future only address water supply volumes and costs, and not wastewater (we have no way to model wastewater futures). This focus on only approximately half the total bill renders our findings about costs relative to income even more striking.

A brief summary of our model of the future this is that we have turned everything in the model into static 2019 real dollars except water supply costs, leaving the rest of the water bill constant with income, a very conservative approach. Water supply costs, in turn, vary only by the structure of volumes supplied and costs of alternative supplies measured in inflation-discounted dollars. These supply costs increase by three to eight times in real 2020 dollars (the year of our data). We then examine the proportion of incomes this one component of water bills will have. The standard of affordability, then, is modest. We focus on the bottom two income quintiles in El Paso Water’s service area. Our approach to estimating the impacts of the rising cost of water on poor households is conservative; we do not favor our key point (that future water will be costly and difficult). Hence, the remarkable figures we present are all the more telling. Ours is a well-reasoned approach to examining the environmental justice effects of the introduction of more costly and difficult water supplies caused by climate change and depletion of cheaper resources.

### 3.8 Correlations with socioeconomic variables

We selected the demographic and socio-economic variables listed in Table 3, based on two sources: the variables examined by Mack and Wrase [6, p8] and variables worth considering in the context of the U.S.-Mexico borderlands and ones providing basic demographic information (percentage population under 18; percentage population over 65; percentage foreign born; percentage non-U.S. citizens; percentage not speaking English at home). We also tabulate the key items used to construct our cost of water model for each tract: mean household income for the five income quintiles in the tract and mean household size by tract.

**Table 3:** Definition of demographic and socio-economic variables used in bivariate regressions.

Variable Name	Explanation
Pct Pop Under 18 years	Percentage of population that is aged 18 years or less
Pct Pop Over 65	Percentage of population that is aged 65 years or more
Pct Pop Disability	Percentage of the civilian non-institutionalized population with a disability
Pct Pop Less than Bachelor’s Degree	Percentage of the population 25 years and over without a bachelor’s degree or higher
Pct HH Female Headed	Percentage of households that are female headed households: no partner present
Pct HH Receiving SNAP	Percentage of households that received food stamps/SNAP in the last 12 months
Pct Pop Foreign Born	Percentage of population either naturalized citizens or non-citizens of the United States
Pct Pop Non US Citizen	Percentage of population not citizens of the United States
Pct Pop Not Speak Only English at Home	Percentage of population that doesn’t only English at home
Pct Rent of HH Gross Income	Median gross rent as a percentage of household income
Pct Civilian Pop Unemployed	Percentage of the civilian population 16 years and older that is unemployed
Pct Pop Black or African American	Percentage of population that is Black
Pct Pop Hispanic or Latino	Percentage of population that is Hispanic
Pct Pop Uninsured	Percentage of the civilian non-institutionalized population without health insurance
Annual Public Assistance Income per capita	Amount of public assistance income received in the last 12 months per capita

Pct = Percentage; Pop = Population; HH = households.

Descriptive statistics, (mean, median, standard deviation, coefficient of variation) are estimated for fraction of households paying 5% or more of income for water supply in our models: the variables in Table 3, quintile annual incomes, and household size. Relationships between fraction of households paying 5% or more of income for water and variables in Table 3 were assessed by performing a series of bivariate, linear regressions. We test for spatial autocorrelation for fraction of households paying 5% or more of income for water, the variables in Table 3, quintile annual incomes, and household size, using Moran’s Index [35], which was estimated with the Global Moran’s I tool in ArcPro. Testing for spatial correlation is important because the regression analysis assumes independence between observations. In the case of variables that are enumerated spatially (e.g., across census tracts), the assumption of independence is violated if there is autocorrelation across a given variable’s spatial distribution. This can lead to loss of model precision, spurious estimates of the goodness of fit of a regression model, and inflated estimates of the effect sizes of independent variables [36].

Lastly, we estimated the correlation matrix for the variables in Table 3 and the lowest quintile mean annual income, to see if there is correlation between the socio-economic variables in Table 3 and between those variables and lowest quintile incomes. Because our primary dependent variable (fraction of households paying 5% or more of income for water) already contains household size and income and the socio-economic variables may tend to correlate with income, correlations between the primary dependent variable and the socio-economic variables may be spurious.

### 3.9 Spatial analysis

We identified spatially-adjacent clusters of census tracts with similar fractions of households in census tracts with a given fraction of income spent on municipal water. We used the cluster analysis tool in ArcGIS Pro, which generates a code indicating where statistically significant clusters of census tracts with high or low levels of fraction of households occur, based on calculations of the Anselin’s Local Moran’s I [37] with associated z-scores and pseudo p-values. The tool generates a code indicating where statistically significant outlier census tracts with of high or low levels of fractions of households within clusters of low or high levels of fractions of households occur. The statistical significance is determined with respect to a 95% confidence level. Our spatial-clustering analysis (discussed below) uses the Climate Change + Imported GW + HB Depletion scenario in 2070, specifically for tracts with households paying over 5% of income in this scenario. But other scenarios and dates represent simple arithmetic transformations of this item, so the geographic patterning remains the same.

## Results and discussion

### 4.1 Future water supply costs

**Fig 4:**
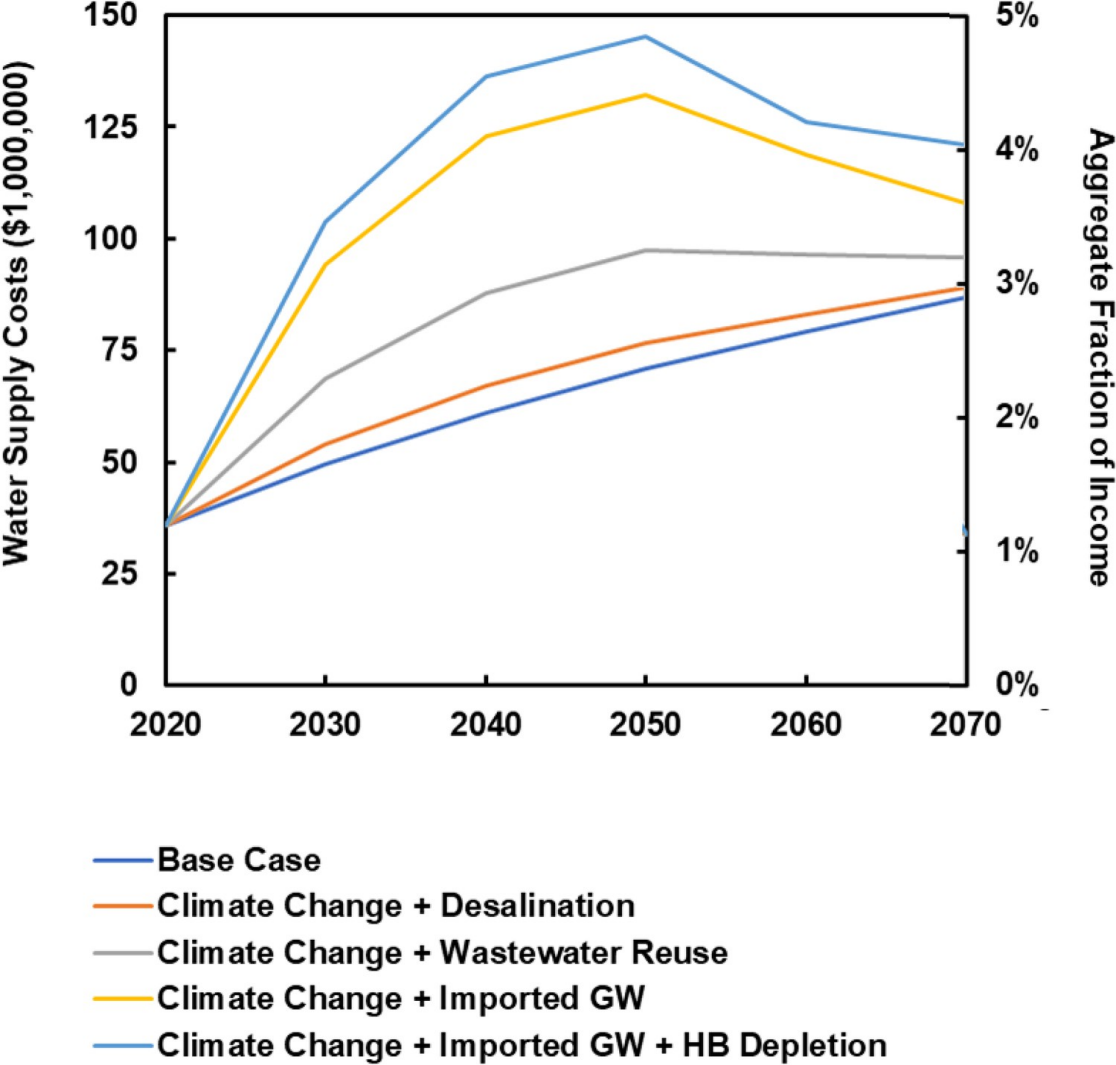
Annual water supply costs and aggregate fraction of income spent on water for service area for each scenario for 2020–2070. Source: Work of the authors.

Fig 4 shows the future water supply costs by year for each water supply scenario. Note that annual costs decrease for the Climate Change + Wastewater Reuse and Climate Change + Imported GW scenarios in 2060 and 2070. This result is due to increased use of cheaper desalination during later years and thus less dependence on more expensive wastewater reuse or imported groundwater. S1 Table in S1 File (see supplemental information) gives the annual water supply volumes for each scenario by water supply source and in total. S2 Table in S1 File (see supplemental information) gives the annual water supply costs for each scenario by water supply source and in total.

### 4.2 Future fractions of income spent on municipal water

**Fig 5:**
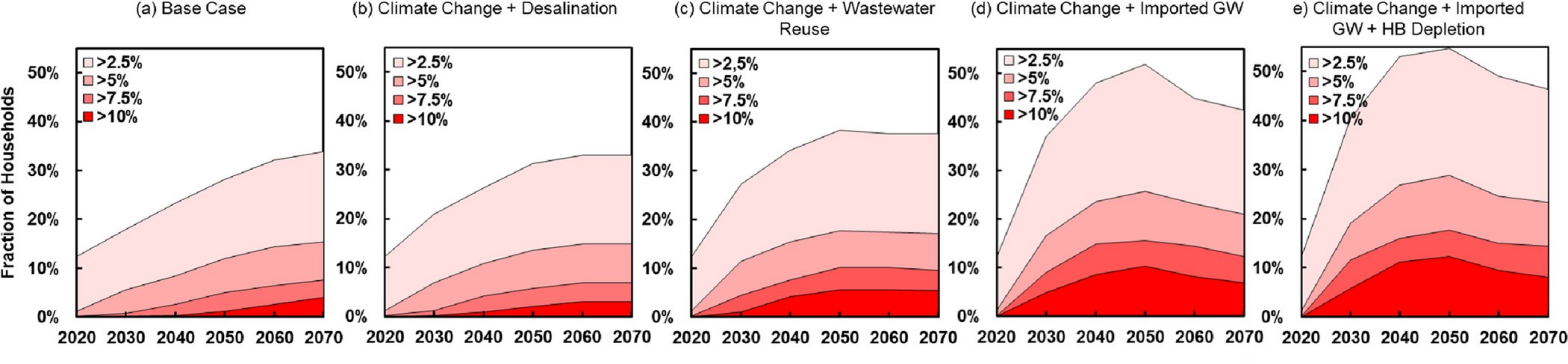
Projected, household-weighted average fraction of population in EPW spending more than a given fraction of income for municipal water over the period 2020–2070 for each water supply scenario. Source: Work of the authors.

Fig 5 shows the projected, household-weighted average fraction of population in EPW spending more than a given fraction of annual income on a basic-needs volume of municipal water over the period 2020–2070 for each water supply scenario. We find significant numbers paying in the future more than 2.5%, and even more than 5% and 7.5%, really large percentages of an income just for minimal quantities of water. The bottom quintile in every future scenario is paying 6% or more of household income by the end of the modeled period. Indeed, we detect notable impacts in the next quintile up, in which basic water supply will be a burden for 40% of all households. The story being told is the same in each case. The poor population in this setting is heavily impacted by the future trajectory of water supply, even when addressing only their basic needs and even when cushioned by a favorable rate structure.

### 4.3 Correlations with socioeconomic variables

Table 4 shows the descriptive statistics for fraction of households paying 5% or more of income for water, the variables in Table 3, quintile annual incomes, and household size. In addition, Table 3 gives the results of the bivariate regressions between fraction of households paying 5% or more of income for water and the variables in Table 3. These results show that most of the bivariate linear regressions are statistically significant, as indicated by relatively high adjusted R^2^ and low p-values for the slopes of the regressions. Most of the slopes are positive, indicating that the fraction of households paying high fractions of income correlates with the potential indicators of vulnerability in Table 3.

**Table 4:** Descriptive statistics and results from bivariate regressions and spatial autocorrelation tests.

Variable	Descriptive Statistics				Bivariate Regression			Spatial Autocorrelation	
	Mean	Median	Standard Deviation	Coefficient of Variation	Adjusted R^2^	Slope	*p*-value for Slope	Moran’s Index	*p*-value for Moran’s Index
Pct HH Paying 5% of Income or Greater	28%	26%	15%	0.55	-	-	-	0.397	< 0.0001
HH Size	2.96	2.99	0.40	0.13	-	-	-	0.156	0.133
Lowest Quintile Mean Annual Income	12,499	11,097	6,500	0.52	-	-	-	0.408	< 0.0001
Second Quintile Mean Annual Income	29,782	27,243	14,270	0.48	-	-	-	0.368	< 0.0001
Third Quintile Mean Annual Income	46,825	43,707	21,446	0.46	-	-	-	0.331	< 0.0001
Fourth Quintile Mean Annual Income	69,909	62,933	29,812	0.43	-	-	-	0.298	< 0.0001
Highest Quintile Mean Annual Income	138,064	122,462	61,582	0.45	-	-	-	0.268	< 0.0001
Pct Pop Under 18 years	25%	25%	6%	0.22	-0.005	-0.125	0.605	0.365	< 0.0001
Pct Pop Over 65	14%	14%	6%	0.41	0.181	1.147	< 0.0001	0.506	< 0.0001
Pct Pop Disability	15%	15%	6%	0.37	0.433	1.845	< 0.0001	0.527	< 0.0001
Pct Pop Less than Bachelor’s Degree	78%	81%	15%	0.19	0.468	1.845	< 0.0001	0.531	< 0.0001
Pct HH Female Headed	32%	32%	8%	0.26	0.381	0.718	< 0.0001	0.297	< 0.0001
Pct HH Receiving SNAP	22%	21%	13%	0.57	0.682	1.007	< 0.0001	0.453	< 0.0001
Pct Pop Foreign Born	25%	24%	8%	0.33	0.387	1.178	< 0.0001	0.453	< 0.0001
Pct Pop Non US Citizen	13%	12%	7%	0.55	0.395	1.318	< 0.0001	0.459	< 0.0001
Pct Pop Not Speak Only English at Home	71%	71%	14%	0.20	0.424	0.698	< 0.0001	0.532	< 0.0001
Pct Rent of HH Gross Income	31%	30%	6%	0.18	0.086	0.826	< 0.0001	0.020	0.481
Pct Civilian Pop Unemployed	7%	6%	4%	0.59	0.219	1.811	< 0.0001	0.111	0.002
Pct Pop Black or African American	4%	3%	4%	1.07	0.048	-0.815	0.006	0.519	< 0.0001
Pct Pop Hispanic or Latino	83%	86%	13%	0.16	0.353	0.699	< 0.0001	0.482	< 0.0001
Pct Pop Uninsured	21%	21%	8%	0.36	0.438	1.360	< 0.0001	0.216	< 0.0001
Annual Public Assistance Income per capita	17	8	25	1.49	0.013	0.001	0.101	0.036	0.242

Pct = Percentage; Pop = Population; HH = households.

The exceptions are Percentage Population Under 18 Years, Percentage Rent of Household Gross Income, Percentage Population Black or African American, and Annual Public Assistance Income per capita. Percentage Population Under 18 years and Percentage Rent of Household Gross Income have relatively narrow distribution compared to the other variables in Table 4 (coefficient of variation = 0.22 and 0.18, respectively), which reduces the effect of these two variables on explaining the variation in fraction of households paying 5% or more of income for water. In El Paso, there are relatively small numbers of Blacks or African Americans (4% of the population in the El Paso Water service area) and the width of the distribution is high (coefficient of variation = 1.07), so the ability of the Percentage Population Black or African American variable to explain variation in fraction of households paying 5% or more of income for water is diminished. Annual Public Assistance Income per capita has a very wide distribution (coefficient of variation = 1.49), which again limits the contribution of this variable towards explaining the variation in fraction of households paying 5% or more of income for water.

The remaining bivariate regressions with the variables appear to be significant in terms of explaining the variation in fraction of households paying 5% or more of income for water. However, the values of the Moran’s I in Table 4 for these variables and the dependent variable (fraction of households paying 5% or more of income for water) are relatively high and the *p*-values are very small. Values of the Moran’s I approaching +0.5 indicate clustering or spatial autocorrelation and low p-values indicate that the hypothesis that values are randomly distributed can be rejected. The Moran’s I values and associated *p*-values show that spatial autocorrelation could produce spurious bivariate regressions. Finally, the correlation matrix (see S4 Table in File) shows relatively high inverse correlations (values < -0.5) between the variables in Table 3 and the lowest quintile income. These high negative correlations imply that there is a strong relationship between the independent variables and income, which is problematic when comparing fraction of households paying 5% or more of income for water, which already has income factored into it, and the socioeconomic variables in Table 3.

To interpret properly these findings, the demographic and socioeconomic variables cannot be treated as independent causal variables, because they are spatially autocorrelated and collinear with income. Income is basic to the construction of the dependent variable. Due to these issues of autocorrelation, apparently significant bivariate relations may be unreliable. Rather, we should understand them as suggesting social characteristics of who is most likely to be impacted by water supply cost increases. Hence, with caution, we suggest that possible characteristics of people impacted will be elevated populations over 65, more disabled, more health-uninsured, more female-headed, more Hispanic, more foreign-born, more non-U.S. citizen, and more not speaking English at home.

### 4.4 Relationships of fractions of income spent on municipal water: Concentrated geographic impacts

**Fig 6:**
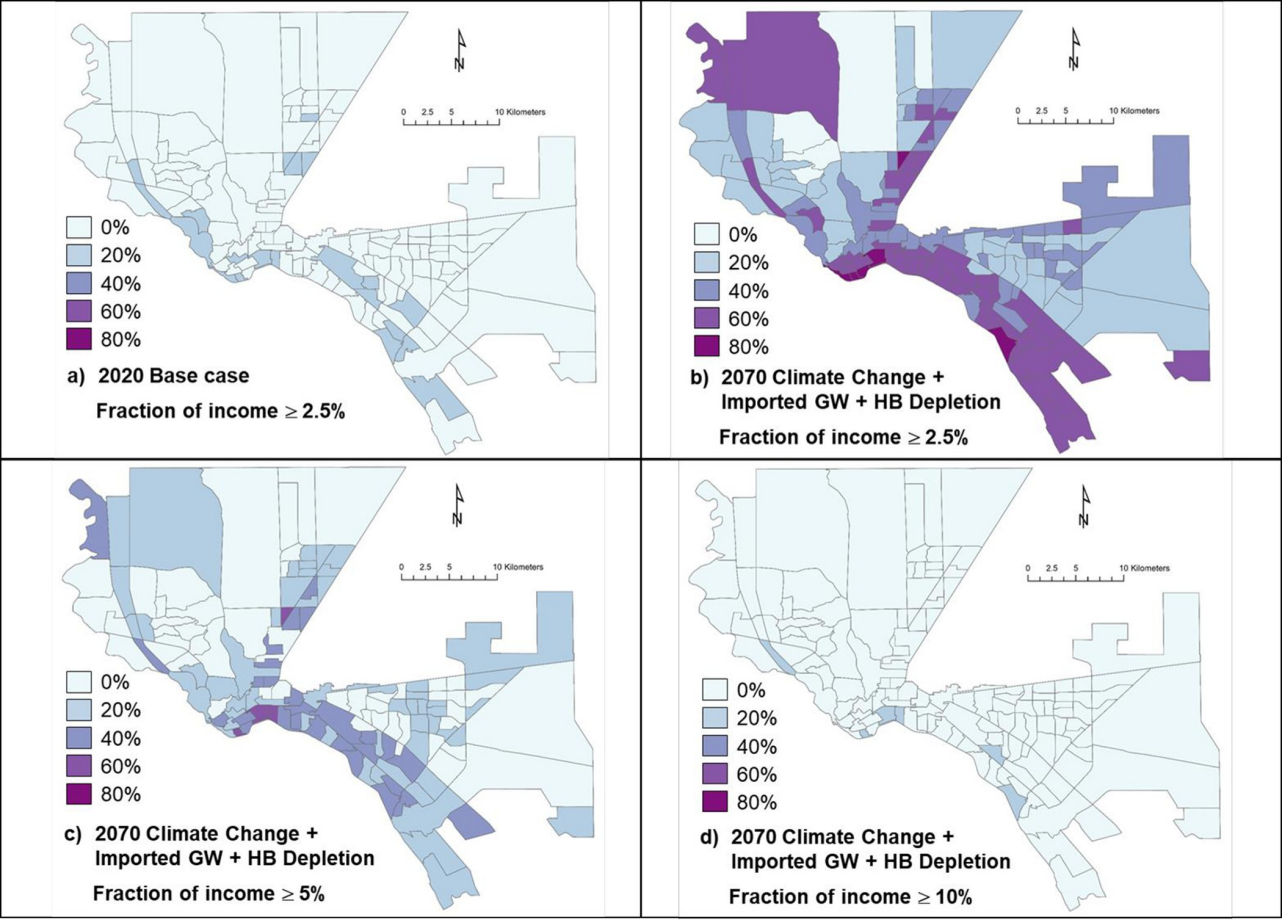
Fractions of households in census tracts (color shading) with fractions of income spent on municipal water of (a) ≥ 2.5% for Base Case and (b) ≥ 2.5%, (c) ≥ 5%, and (d) ≥ 10% for the Climate Change + Imported GW + HB Depletion scenario in 2070. Basemap (census tract boundaries): TIGER Geodatabase, US Census. Data representation: Work of the authors.

**Fig 7:**
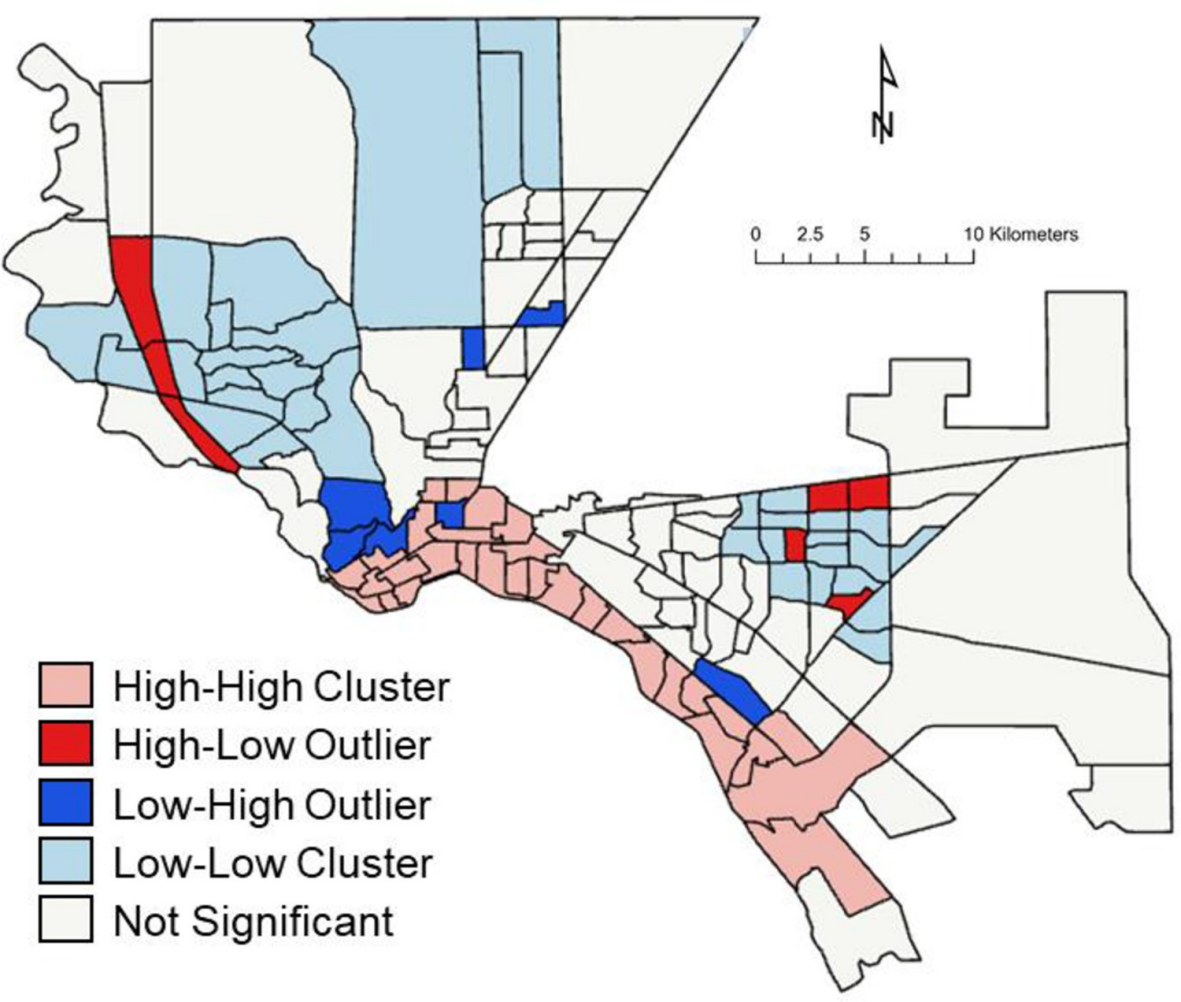
Statistically-significant (95% confidence level) clusters and outliers for fractions of households in census tracts with fractions of income spent on municipal water of ≥ 5%,for the Climate Change + Imported GW + HB Depletion scenario in 2070. High-High Cluster and Low-Low Cluster refer to clusters of census tracts with high or levels of fractions of households, respectively. High-Low Outlier and Low-High Outlier refer to census tracts with high or low levels of fractions of households located in clusters of low or high levels of fractions of households, respectively. Not Significant refers to census tracts that are neither part of a statistically significant cluster nor a statistically significant outlier, relative to the 95% confidence level. Basemap (census tract boundaries): TIGER Geodatabase, US Census. Data representation: Work of the authors.

The impact is unequally distributed. Visually, Fig 6 shows the distribution by census tract, of households in census tracts with fractions of income spent on municipal water of (a) ≥ 2.5%, (b) ≥ 5%, and (c) ≥ 10% for the Climate Change + Imported GW + HB Depletion scenario in 2070. We see a notable pattern of impacted households in poor tracts in the south and the “northeast” areas of the city (northeast is the local term for the arm of the city extending north between the Franklin Mountains and Ft. Bliss). We calculated the Moran index for each of the mappings of fraction of income and found mean values of Moran’s Index of 0.133, a z-score of 4.05 and a p-value of < 0.0001. These results indicate that there is substantial spatial clustering. To explore this further, we used an ArcGIS tool for measuring high and low clusters (see methods, above). Fig 7, pink color, shows a clear clustering of cluster of tracts and their neighbors facing expenses over 5% along the Mexican border (areas of the city that largely are poor, Hispanic, Spanish speaking, and immigrant, though not uniquely so in the case of El Paso). Red also shows tracts with elevated cost impacts, but ones that differ from neighbors with low impacts. Our modeling and analysis suggest an important concern with the geographically disproportionate, environmental justice impact of future costly water.

### 4.5 Study limitations

Limitations of this work include reliance on other projections and studies to model the future. These include relying on Texas Water Development Board projections of regional population and water demand which could be affected by many factors; reliance on El Paso Water’s projected water infrastructure/technology costs, which can change; use of the moderately dry downscaled climate change model for river input to the region, which could be wetter or drier; and use of our research team’s water balance model, which is more pessimistic about the future of the freshwater in the Hueco Bolson than the Texas Water Development Board is (however, every model projects freshwater exhaustion of that aquifer in this century, under current usage scenarios). We focus on a basic need level of water consumption, so we did not examine the price impacts of future water supplies at higher usage levels by actual poor and middle-income users in El Paso. To simplify, we assumed that higher temperatures and lengthening of the cooling season will not change basic needs water, while likely there will be some increase via evaporative coolers. We used the current water billing structure, which could change. This is a notably conservative assumption vis-à-vis our argument, because EPW’s current structure favors the poor.

A data limitation is that the socio-economic data is taken from the American Community Survey (ACS) of the U.S. Census. The ACS reports a best estimate within a 90% confidence interval for census tracts. Future studies can explore the impact of this and other uncertainties.

An important limitation is that we do not incorporate price-demand responses in our analysis. As in most municipalities, water consumption in El Paso follows an inverse and inelastic relationship with price [38]. However, we did not have confidence in applying price-demand relationships because that would assume that drivers of water use would remain constant 50 years into the future. Furthermore, we do not know how household incomes and expenses other than water will change over that period. We do note that Fullerton et al. [38] found an elasticity of -0.32 (a 10% rate increase would produce a 3.2% decrease in water demand) for monthly water use in El Paso. They also found that, at least for monthly water use, water consumers tend to react more quickly to changes in climatic conditions than to changes in price, which could dampen the impact of price on reducing demand and heighten the impact of expected increases in temperature. Our estimates of increased costs for water assume that the basic need volume for poor consumers remains constant over the 50-year period, that is, there are limited ways to survive without basic water.

The majority of the literature reports that residential water demand, in general, is price inelastic, with few studies reporting price elasticity estimates larger than -0.25. Meta analyses have reported mean elasticities of -0.51 [39], -0.37 [40], and -0.34 [41]. Capt [42] reports an elasticity of -0.30 for El Paso, which is on the high end of these estimates (also see Fullerton et al. [38]). Demand and price elasticities are sensitive to weather and seasonal factors. Summer water demands can be substantially higher than winter use due to increases in outdoor water use. However, summer price elasticities are usually larger than winter ones, since discretionary water uses, such as outdoor use, are more price-sensitive than non-discretionary uses, such as indoor uses [40,41,43–45]. For example, Marzano et al. [40] estimated winter and summer price elasticities as -0.26 and -0.59, respectively, in their meta-analysis. Mansur et al. [46] found indoor and outdoor price elasticities of -0.093 and -0.618, respectively. All of these studies imply that the response to price increases of households in our study will be minimal, since our basic use figure is explicitly for indoor use [31 p33]. The exception is our inclusion of evaporative cooler use, which is primarily a summer use. While no studies of the elasticity of evaporative cooler use to water price appear in the literature, we consider that evaporative cooler use tends to be non-discretionary in a hot but normally dry environment.

While overall consumption likely would decrease with price increases (slowing the introduction of expensive alternatives), the basic needs amount of water likely will be inelastic, making that aspect of our analysis less affected by this limitation. Overall, we recognize that future models for predicting the impact of water scarcity on the poor should account for price increases and potential corresponding decreases in water demand.

## Conclusion

The current literature identifies cost of water as a stress on poor consumers [6,9,11–13]. Past national trends show increases in water cost faster than incomes and the overall consumer price index [1–4]. However, literature on water cost stress has no or limited tools to model the future except a projection of recent existing trends (see our discussion above of Mack and Wrase [6], Colton [9]). Linear projection does not address major systemic changes. Teodoro [12] who offers a convincing current measure of impact of water prices on the poorest people in a number of cities, identifies El Paso as among the cities having the least impact on the poor. Teodoro is likely correct for the present, but we find forces, such as climate change and groundwater depletion, as drivers of major cost increases likely in the future. Our contribution to the literature, then, is to add more robust and detailed models that link regional climate change and resource exploitation transitions to water cost impact on the poor.

We provide a possibly transportable model for how to project water cost futures over a long period of time (50 years), addressing major socio-hydrological transitions and not just linearly projecting the recent past. Transitions involve significant rearrangement of socio-natural processes over time to create and adjust to directional change [47–50]. This theme has drawn interest recently in the context of climate change and change toward a greener economy, taking note of disruptive as well as transformative effects. The literature on the social effects of transitions has concentrated on resource extraction dependent communities. We argue that vulnerable consumers also need to be considered in the transitions literature. In this study, we trace a possible sequence of changes starting from climate change affecting the river supply and depletion of the cheapest, easiest alternative, near surface fresh groundwater, on residential water costs. We include alternative scenarios for climate effects, tracing the volumes of water supply that would need to be replaced, based on a comprehensive regional water budget model. The next step is assembling evidence from planning documents about the costs and volumes of alternative new sources of water, including notable options such as long-distance importation, desalination, and direct potable reuse; these are handled as multiple supply scenarios. The outcome seen in all scenarios is that to fulfill regional demand in the future will require more distant sources and/or complex methods of processing water of poorer quality. These responses to water scarcity, not surprisingly, will be much more expensive than current costs of providing water. We thus make a case for a key conceptual proposition, that future residential water will be more costly and difficult.

We cannot predict who will pay for more costly future water, but the simplest assumption is that water costs will increase significantly, as just described, but will be distributed exactly as it is now, with low volume consumers subsidized (via pricing structure) by high volume consumers. Even so, consumers of low volumes will face steep price increases. We thus broach—but do not answer—an important set of policy questions about how impending price increases will be distributed.

The U.S. Census provides valuable information on the distribution of ability to pay, permitting us to delineate the possible impact of future cost increases. If we examine a basic minimum amount of water for indoor use and simple evaporative cooling in summer, uses people can ill afford to sacrifice, we find large numbers of people who will have to pay notably large amounts of money for water supply. In El Paso, this is geographically unequal, so we can identify likely zones of concentrated impact. Furthermore, while El Paso is so heavily Hispanic that our finding of concentrated possible price impacts on poor Hispanics is uninteresting, it is worth positing that in the U.S. west and southwest we can reasonably predict a price risk specifically for poor Hispanics. We thus delineate a causal path of climate change impact on Hispanics, along with negative impacts from resource (fresh groundwater) depletion and increasing populations in the region.

These findings join an important recent literature on the impact of water cost on vulnerable people even in the global north with supposedly good residential water supplies [51]. To it, we add a well-reasoned way of building and examining scenarios about future changes. Costly and difficult water will negatively impact the poor and people of color. Our current field research addresses the challenge current utility bills present to the very poor, to understand the baseline for and possible impacts of these likely future changes. Our work thus adds to the expanding literature on household water insecurity [10].

## Supporting information

S1 File S1 Table. Annual volumes for each water source by scenario and by year (AF = acre feet). **S2 Table.** Annual costs for each water source by scenario and by year. **S3 Table.** Census tract codes (El Paso County, Texas). **S4 Table**. Correlation Matrix.
(DOCX)
