# Supplementary material for: Predictions of household water affordability under conditions of climate change, demographic growth, and fresh groundwater depletion in a southwest US city indicate increasing burdens on the poor
Source: PLoS One. 2022 Nov 23;17(11):e0277268. doi: 10.1371/journal.pone.0277268 (PMC9683544; doi:10.1371/journal.pone.0277268)
Supplement: S1 File — Annual volumes for each water source by scenario and by year (AF = acre feet). S2 Table. Annual costs for each water source by scenario and by year. S3 Table. Census tract codes (El Paso County, Texas). S4 Table. Correlation Matrix. (DOCX) [file pone.0277268.s001.docx]

Supporting Information for

**Predictions of household water affordability under conditions of climate change, demographic growth, and fresh groundwater depletion in a southwest US city indicate increasing burdens on the poor and Hispanics**

Josiah Heyman, University of Texas at El Paso, El Paso, Texas, USA

Alex Mayer, University of Texas at El Paso, El Paso, Texas, USA

Jessica Alger, Michigan Technological University, Houghton, Michigan, USA

**Contents of this file**

Introduction

Tables S1 to S4

Shapefiles for El Paso Water utility service boundaries

**Introduction**

The Supporting Information contains supplemental tables of annual volumes and costs for each water supply source and by year for each scenario, the census tract numbers used in the analysis of incomes spent on water, and shapefiles for the El Paso Water utility service area boundaries.

Projected water supply availability volumes for 2020 to 2070 were taken from the 2016 Far West Texas Water Plan (FWTWP) (Texas Water Development Board, TWDB, 2016). The FWTWP projects future water supply availability from existing sources, plans for new sources, and reductions in per capita demand, to compensate for the deficit between projected demand and current water supply availability. However, we suggest that the FWTWP estimates of water availability from two primary current sources, Hueco Bolson freshwater and the Rio Grande, are overly optimistic. First, given current freshwater pumping rates from the Hueco Bolson aquifer by El Paso, Ciudad Juárez, and agricultural irrigators (approximately 188,000 AF/yr together), estimates of aquifer recharge rates (approximately 33,000 AF/yr), and remaining freshwater storage in the aquifer (approximately 6,500,000 AF), freshwater is expected to be completely depleted in 42 years (Mayer et al., in review). It is likely that pumping rates in Ciudad Juárez will increase substantially in the meantime, as populations in this city may increase as much as 1.02% per year, for a total increase of 66% by 2070. Given current aquifer recharge rates and expected increases in pumping by Ciudad Juárez, we estimate, using our water balance model (https://swim.cybershare.utep.edu/en/wb-intro), that EPW’s current rate of pumping of freshwater from the Hueco Bolson will need to be reduced from the amounts predicted in the FWTWP by 50% by 2070 to extend the aquifer life past 2070.

Second, climate change in the headwaters of the Rio Grande are expected to substantially decrease downstream supplies, with flows into Elephant Butte reservoir declining 5%, averaged across a series of climate change scenarios (Townsend and Gutzler, 2020). Using a pessimistic climate change scenario associated with the Access85 climate model projection, inflows into Elephant Butte will decline by 39% on average over the period 2020-2070, effectively reducing average water availability for EPW from the Rio Grande by the same fraction. Using projected annual Elephant Butte reservoir inflows from Townsend and Gutzler (2020) for the Access85 climate model projection, the declines in Rio Grande water availability by year will be 37%, 56%, 60%, 41%, and 23% in the years 2030, 2040, 2050, 2060, and 2070, respectively. These declines were calculated using the water balance model.

Four scenarios for future supply volumes by source were determined (Table S1) based on the expected impacts of climate change and groundwater depletion and how these decreases will be compensated by increases in desalinated Hueco Bolson brackish groundwater versus imported groundwater. While depletion of Hueco Bolson fresh groundwater is expected to occur in a matter of a few decades, less is known about the availability of Hueco Bolson brackish groundwater over the next 50 years. To contend with this uncertainty, we incorporate into the scenarios the possibility that reductions in Rio Grande supply and Hueco Bolson freshwater pumping will be compensated by either 100% from desalinated Hueco Bolson brackish groundwater or 100% from imported groundwater.

The four water supply scenarios are: (1) “Base Case,” which is based the FWTWP expectations for meeting supply deficits; (2) “Climate Change + Desalination,” in which the Rio Grande water supply is reduced according to the Access85 climate model predictions and the reduction is compensated by increasing desalinated brackish groundwater from the Hueco Bolson; (3) “Climate Change + Imported GW,” in which the Rio Grande water supply is reduced according to the Access85 climate model predictions and the reduction is compensated by increasing imported groundwater; and (4) “Climate Change + Imported GW + Reduction in HB Pumping,” in which the Rio Grande water supply is reduced according to the Access85 climate model predictions, freshwater pumping from the Hueco Bolson (HB) is reduced by 50%, and the two supply reductions are compensated by increasing imported groundwater. Table S1 in the supplementary information shows the annual volumes for each water source by scenario and by year.

The 2016 FWTWP identifies costs associated with expansion of water supply sources. However, we use updated costs of sources directly obtained from EPW (Lisa Franklin Rosendorf, personal communication). These unit costs are cost of supply in 2020 per volume of water supplied and are assumed to include amortized capital and operation and maintenance (O&M) costs. Interest rates for bonds from TWDB to pay for capital water supply improvements are usually low, on the order of 2-3% over the lifetime of the bond. We do not inflate the unit costs from 2020 to 2070, because we take the approach involving the simplest possible assumptions that inflation in unit water supply costs will be matched by increases in household incomes, effectively canceling out increases in water supply costs due to inflation. See Table S2.

The Census Tract numbers in Table S3 are for all of the census tract within the El Paso Water service area boundary and relate to the tract numbers used in the 2020 US Census and American Community Survey. The census tracts within the El Paso Water service area boundary were determined in ArcGIS by overlaying a layer of the service area boundaries on a layer of US census tracts in El Paso County and applying a join tool. All census tracts with overlap of at least 15% were selected. The shapefiles for the service area boundaries are included in this data package in the folder “boundary11.24.21.gdb.”

**Table S1.** Annual volumes for each water source by scenario and by year (AF = acre feet)

| Base Case |  | |  | |  | |  | |  | |  | |
| --- | --- | --- | --- | --- | --- | --- | --- | --- | --- | --- | --- | --- |
| Supply by source (AF) | | |  | |  | |  | |  | |  | |
|  | 2020 | | 2030 | | 2040 | | 2050 | | 2060 | | 2070 | |
| Mesilla GW | | 26860 | | 26948 | | 27036 | | 27124 | | 27212 | | 27300 |
| Hueco GW | | 60040 | | 56852 | | 53664 | | 50476 | | 47288 | | 44100 |
| Rio Grande | | 63200 | | 64000 | | 64800 | | 65600 | | 66400 | | 67200 |
| Desal | | 7900 | | 10520 | | 13140 | | 15760 | | 18380 | | 21000 |
| Imported GW | | 0 | | 5460 | | 10920 | | 16380 | | 21840 | | 27300 |
| AWPF+ASR | | 0 | | 4620 | | 9240 | | 13860 | | 18480 | | 23100 |
| Total | | 158000 | | 168400 | | 178800 | | 189200 | | 199600 | | 210000 |
|  |  | |  | |  | |  | |  | |  | |
| Access RG + makeup with Desal | | | | |  | |  | |  | |  | |
| Supply by source (AF) | | |  | |  | |  | |  | |  | |
|  | 2020 | | 2030 | | 2040 | | 2050 | | 2060 | | 2070 | |
| Mesilla GW | | 26860 | | 26948 | | 27036 | | 27124 | | 27212 | | 27300 |
| Hueco GW | | 60040 | | 56852 | | 53664 | | 50476 | | 47288 | | 44100 |
| Rio Grande | | 63200 | | 40320 | | 28512 | | 26240 | | 39176 | | 51744 |
| Desal | | 7900 | | 34200 | | 49428 | | 55120 | | 45604 | | 36456 |
| Imported GW | | 0 | | 5460 | | 10920 | | 16380 | | 21840 | | 27300 |
| AWPF+ASR | | 0 | | 4620 | | 9240 | | 13860 | | 18480 | | 23100 |
| Total | | 158000 | | 168400 | | 178800 | | 189200 | | 199600 | | 210000 |
|  |  | |  | |  | |  | |  | |  | |
| Access RG + makeup with Imported GW | | | | | | |  | |  | |  | |
| Supply by source (AF) | | |  | |  | |  | |  | |  | |
|  | 2020 | | 2030 | | 2040 | | 2050 | | 2060 | | 2070 | |
| Mesilla GW | | 26860 | | 26948 | | 27036 | | 27124 | | 27212 | | 27300 |
| Hueco GW | | 60040 | | 56852 | | 53664 | | 50476 | | 47288 | | 44100 |
| Rio Grande | | 63200 | | 40320 | | 28512 | | 26240 | | 39176 | | 51744 |
| Desal | | 7900 | | 10520 | | 13140 | | 15760 | | 18380 | | 21000 |
| Imported GW | | 0 | | 29140 | | 47208 | | 55740 | | 49064 | | 42756 |
| AWPF+ASR | | 0 | | 4620 | | 9240 | | 13860 | | 18480 | | 23100 |
| Total | | 158000 | | 168400 | | 178800 | | 189200 | | 199600 | | 210000 |

| Access RG + makeup with AWPF+ASR | | | | | | |  | |  | |  | |
| --- | --- | --- | --- | --- | --- | --- | --- | --- | --- | --- | --- | --- |
| Supply by source (AF) | | |  | |  | |  | |  | |  | |
|  | 2020 | | 2030 | | 2040 | | 2050 | | 2060 | | 2070 | |
| Mesilla GW | | 26860 | | 26948 | | 27036 | | 27124 | | 27212 | | 27300 |
| Hueco GW | | 60040 | | 56852 | | 53664 | | 50476 | | 47288 | | 44100 |
| Rio Grande | | 63200 | | 40320 | | 28512 | | 26240 | | 39176 | | 51744 |
| Desal | | 7900 | | 10520 | | 13140 | | 15760 | | 18380 | | 21000 |
| Imported GW | | 0 | | 5460 | | 10920 | | 16380 | | 21840 | | 27300 |
| AWPF+ASR | | 0 | | 28300 | | 45528 | | 53220 | | 45704 | | 38556 |
| Total | | 158000 | | 168400 | | 178800 | | 189200 | | 199600 | | 210000 |
|  |  | |  | |  | |  | |  | |  | |
| Access RG + 50% decrease in HB + makeup with Imported GW | | | | | | | | | | |  | |
| Supply by source (AF) | | |  | |  | |  | |  | |  | |
|  | 2020 | | 2030 | | 2040 | | 2050 | | 2060 | | 2070 | |
| Mesilla GW | | 26860 | | 26948 | | 27036 | | 27124 | | 27212 | | 27300 |
| Hueco GW | | 60040 | | 51167 | | 42931 | | 35333 | | 28373 | | 22050 |
| Rio Grande | | 63200 | | 40320 | | 28512 | | 26240 | | 39176 | | 51744 |
| Desal | | 7900 | | 34200 | | 49428 | | 55120 | | 45604 | | 36456 |
| Imported GW | | 0 | | 29140 | | 47208 | | 55740 | | 49064 | | 49350 |
| AWPF+ASR | | 0 | | 4620 | | 9240 | | 13860 | | 18480 | | 23100 |
| Total | | 158000 | | 186395 | | 204355 | | 213417 | | 207909 | | 210000 |

**Table S2.** Annual costs for each water source by scenario and by year

| Base Case |  |  | |  | | |  | | |  | | |  | |  |
| --- | --- | --- | --- | --- | --- | --- | --- | --- | --- | --- | --- | --- | --- | --- | --- |
| Cost by source ($1,000) |  |  | | | |  | | |  | | |  | | | |
|  | 2020 | | 2030 | | 2040 | | | 2050 | | | 2060 | | | 2070 | |
| Mesilla GW | 4,029 | | 4,042 | | 4,055 | | | 4,069 | | | 4,082 | | | 4,095 | |
| Hueco GW | 9,006 | | 8,528 | | 8,050 | | | 7,571 | | | 7,093 | | | 6,615 | |
| Rio Grande | 18,960 | | 19,200 | | 19,440 | | | 19,680 | | | 19,920 | | | 20,160 | |
| Desal | 3,950 | | 5,260 | | 6,570 | | | 7,880 | | | 9,190 | | | 10,500 | |
| Imported GW | - | | 13,104 | | 26,208 | | | 39,312 | | | 52,416 | | | 65,520 | |
| AWPF+ASR | - | | 5,544 | | 11,088 | | | 16,632 | | | 22,176 | | | 27,720 | |
| Total | 35,945 | | 55,678 | | 75,411 | | | 95,144 | | | 114,877 | | | 134,610 | |
|  |  |  | |  | | |  | | |  | | |  | |  |
| Access RG + makeup with Desal | | | |  | | |  | | |  | | |  | |  |
| Cost by source ($1,000) | |  | |  | | |  | | |  | | |  | |  |
|  | 2020 | 2030 | | 2040 | | | 2050 | | | 2060 | | | 2070 | |  |
| Mesilla GW | 4,029 | 4,042 | | 4,055 | | | 4,069 | | | 4,082 | | | 4,095 | |  |
| Hueco GW | 9,006 | 8,528 | | 8,050 | | | 7,571 | | | 7,093 | | | 6,615 | |  |
| Rio Grande | 18,960 | 12,096 | | 8,554 | | | 7,872 | | | 11,753 | | | 15,523 | |  |
| Desal | 3,950 | 17,100 | | 24,714 | | | 27,560 | | | 22,802 | | | 18,228 | |  |
| Imported GW | - | 13,104 | | 26,208 | | | 39,312 | | | 52,416 | | | 65,520 | |  |
| AWPF+ASR | - | 5,544 | | 11,088 | | | 16,632 | | | 22,176 | | | 27,720 | |  |
| Total | 35,945 | 60,414 | | 82,669 | | | 103,016 | | | 120,322 | | | 137,701 | |  |
|  |  |  | |  | | |  | | |  | | |  | |  |

**Table S2.** Annual costs for each water source by scenario and by year, continued

| Access RG + 50% decrease in HB + makeup with Imported GW | | | | | | | | | |  |
| --- | --- | --- | --- | --- | --- | --- | --- | --- | --- | --- |
| Cost by source ($1,000) |  | |  | |  | |  | |  | |
|  | 2020 | 2030 | | 2040 | | 2050 | | 2060 | | 2070 |
| Mesilla GW | 4,029 | 4,042 | | 4,055 | | 4,069 | | 4,082 | | 4,095 |
| Hueco GW | 9,006 | 7,675 | | 6,440 | | 5,300 | | 4,256 | | 3,308 |
| Rio Grande | 18,960 | 12,096 | | 8,554 | | 7,872 | | 11,753 | | 15,523 |
| Desal | 3,950 | 17,100 | | 24,714 | | 27,560 | | 22,802 | | 18,228 |
| Imported GW | - | 69,936 | | 113,299 | | 133,776 | | 117,754 | | 118,440 |
| AWPF+ASR | - | 5,544 | | 11,088 | | 16,632 | | 22,176 | | 27,720 |
| Total | 35,945 | 116,393 | | | 168,150 | 195,209 | | 182,822 | | 187,314 |

**Table S3.** Census tract codes (El Paso County, Texas)

| Census Tract 1.01 | Census Tract 11.13 | Census Tract 34.04 | Census Tract 43.16 | Census Tract 103.28 |
| --- | --- | --- | --- | --- |
| Census Tract 1.06 | Census Tract 11.14 | Census Tract 35.01 | Census Tract 43.17 | Census Tract 103.29 |
| Census Tract 1.07 | Census Tract 11.15 | Census Tract 35.02 | Census Tract 43.18 | Census Tract 103.30 |
| Census Tract 1.08 | Census Tract 12.01 | Census Tract 36.01 | Census Tract 43.19 | Census Tract 103.31 |
| Census Tract 1.09 | Census Tract 12.02 | Census Tract 36.02 | Census Tract 43.20 | Census Tract 103.33 |
| Census Tract 1.10 | Census Tract 12.03 | Census Tract 37.01 | Census Tract 102.07 | Census Tract 103.36 |
| Census Tract 1.11 | Census Tract 13.01 | Census Tract 37.02 | Census Tract 102.10 | Census Tract 103.37 |
| Census Tract 1.12 | Census Tract 13.02 | Census Tract 38.01 | Census Tract 102.11 | Census Tract 103.38 |
| Census Tract 2.04 | Census Tract 14 | Census Tract 38.03 | Census Tract 102.12 | Census Tract 103.39 |
| Census Tract 2.05 | Census Tract 15.01 | Census Tract 38.04 | Census Tract 102.13 | Census Tract 103.41 |
| Census Tract 2.06 | Census Tract 15.02 | Census Tract 39.01 | Census Tract 102.14 | Census Tract 103.42 |
| Census Tract 2.07 | Census Tract 16 | Census Tract 39.02 | Census Tract 102.15 | Census Tract 103.46 |
| Census Tract 2.08 | Census Tract 17 | Census Tract 39.03 | Census Tract 102.16 | Census Tract 104.01 |
| Census Tract 3.01 | Census Tract 18 | Census Tract 40.02 | Census Tract 102.17 | Census Tract 104.05 |
| Census Tract 3.02 | Census Tract 19 | Census Tract 40.03 | Census Tract 102.18 | Census Tract 106 |
| Census Tract 4.01 | Census Tract 20 | Census Tract 40.04 | Census Tract 102.19 |  |
| Census Tract 4.03 | Census Tract 21 | Census Tract 41.03 | Census Tract 102.20 |  |
| Census Tract 4.04 | Census Tract 22.01 | Census Tract 41.04 | Census Tract 102.21 |  |
| Census Tract 6 | Census Tract 22.02 | Census Tract 41.05 | Census Tract 102.22 |  |
| Census Tract 8 | Census Tract 23 | Census Tract 41.06 | Census Tract 103.03 |  |
| Census Tract 9 | Census Tract 24 | Census Tract 41.07 | Census Tract 103.07 |  |
| Census Tract 10.01 | Census Tract 25 | Census Tract 42.01 | Census Tract 103.11 |  |
| Census Tract 10.02 | Census Tract 26 | Census Tract 42.02 | Census Tract 103.12 |  |
| Census Tract 11.04 | Census Tract 28 | Census Tract 43.03 | Census Tract 103.16 |  |
| Census Tract 11.07 | Census Tract 29 | Census Tract 43.07 | Census Tract 103.17 |  |
| Census Tract 11.09 | Census Tract 30 | Census Tract 43.09 | Census Tract 103.22 |  |
| Census Tract 11.10 | Census Tract 31 | Census Tract 43.10 | Census Tract 103.23 |  |
| Census Tract 11.11 | Census Tract 32 | Census Tract 43.11 | Census Tract 103.24 |  |
| Census Tract 11.12 | Census Tract 33 | Census Tract 43.12 | Census Tract 103.25 |  |

Table S4. Correlation Matrix

|  | Pct Pop Under 18 years | Pct Pop Over 65 | Pct Pop Disability % | Pct Pop Less than Bachelor's degree | Pct HH Female Headed | Pct HH Receiving SNAP | Pct Pop Foreign Born | Pct Pop Non US Citizen | Pct Pop Not Speak Only English at Home | Pct Rent of HH Gross Income | Pct Civilian Pop Unemployed | Pct Pop Black or African American | Pct Pop Hispanic or Latino | Pct Pop Uninsured | Annual Public Assistance Income per capita | Lowest Quintile |
| --- | --- | --- | --- | --- | --- | --- | --- | --- | --- | --- | --- | --- | --- | --- | --- | --- |
| Pct Pop Under 18 years | 1.000 |  |  |  |  |  |  |  |  |  |  |  |  |  |  |  |
| Pct Pop Over 65 | -0.534 | 1.000 |  |  |  |  |  |  |  |  |  |  |  |  |  |  |
| Pct Pop Disability % | -0.328 | 0.655 | 1.000 |  |  |  |  |  |  |  |  |  |  |  |  |  |
| Pct Pop Less than Bachelor's degree | 0.028 | 0.283 | 0.657 | 1.000 |  |  |  |  |  |  |  |  |  |  |  |  |
| Pct HH Female Headed | -0.267 | 0.447 | 0.563 | 0.507 | 1.000 |  |  |  |  |  |  |  |  |  |  |  |
| Pct HH Receiving SNAP | 0.001 | 0.365 | 0.704 | 0.779 | 0.638 | 1.000 |  |  |  |  |  |  |  |  |  |  |
| Pct Pop Foreign Born | -0.328 | 0.329 | 0.535 | 0.452 | 0.382 | 0.659 | 1.000 |  |  |  |  |  |  |  |  |  |
| Pct Pop Non US Citizen | -0.266 | 0.249 | 0.530 | 0.466 | 0.380 | 0.695 | 0.930 | 1.000 |  |  |  |  |  |  |  |  |
| Pct Pop Not Speak Only English at Home | -0.201 | 0.369 | 0.586 | 0.639 | 0.487 | 0.694 | 0.772 | 0.686 | 1.000 |  |  |  |  |  |  |  |
| Pct Rent of HH Gross Income | 0.114 | 0.114 | 0.166 | 0.162 | 0.231 | 0.319 | 0.171 | 0.202 | 0.179 | 1.000 |  |  |  |  |  |  |
| Pct Civilian Pop Unemployed | 0.069 | 0.180 | 0.282 | 0.380 | 0.353 | 0.468 | 0.183 | 0.248 | 0.203 | 0.139 | 1.000 |  |  |  |  |  |
| Pct Pop Black or African American | 0.130 | -0.307 | -0.115 | -0.063 | -0.173 | -0.179 | -0.355 | -0.249 | -0.589 | -0.122 | 0.015 | 1.000 |  |  |  |  |
| Pct Pop Hispanic or Latino | -0.075 | 0.308 | 0.501 | 0.664 | 0.438 | 0.647 | 0.609 | 0.535 | 0.914 | 0.193 | 0.176 | -0.643 | 1.000 |  |  |  |
| Pct Pop Uninsured | -0.190 | 0.179 | 0.477 | 0.653 | 0.500 | 0.706 | 0.685 | 0.717 | 0.654 | 0.178 | 0.332 | -0.234 | 0.614 | 1.000 |  |  |
| Annual Public Assistance Income per capita | 0.100 | 0.024 | 0.082 | 0.183 | 0.174 | 0.204 | -0.014 | 0.014 | 0.010 | 0.151 | 0.125 | 0.107 | 0.046 | 0.053 | 1.000 |  |
| Lowest Quintile Annual Average Income | 0.200 | -0.401 | -0.545 | -0.572 | -0.666 | -0.700 | -0.496 | -0.542 | -0.532 | -0.349 | -0.377 | 0.181 | -0.510 | -0.643 | -0.115 | 1.000 |
